# Conduits for Right Ventricular Outflow Tract Reconstruction in Infants and Young Children

**DOI:** 10.3389/fsurg.2021.719840

**Published:** 2021-09-22

**Authors:** Tao Qian, Haoyong Yuan, Chunyang Chen, Yuhong Liu, Ting Lu, Can Huang, Zhongshi Wu

**Affiliations:** ^1^Department of Cardiovascular Surgery, The Second Xiangya Hospital, Central South University, Changsha, China; ^2^Engineering Laboratory of Hunan Province for Cardiovascular Biomaterials, Changsha, China

**Keywords:** right ventricular outflow react reconstruction, valved conduit, children, homograft, bovine jugular vein conduit, expanded polytetrafluoroethylene, tissue engineering

## Abstract

**Purpose of Review:** Right ventricular outflow tract (RVOT) reconstruction remains a challenge due to the lack of an ideal conduit. Data and experience are accumulating with each passing day. Therefore, it is necessary to review this topic from time to time. This is a 2021 update review focused on the history, evolution, and current situation of small-sized conduits (≤ 16 mm) for RVOT reconstruction in infants and young children.

**Recent Findings:** Currently, the available small-sized (≤16 mm) conduits can meet most clinical needs. Homograft is still a reliable choice for infants and young children validated by a half-century clinical experience. As an alternative material, bovine jugular vein conduit (BJVC) has at least comparable durability with that of homograft. The performance of expanded polytetrafluoroethylene (ePTFE) is amazing in RVOT position according to limited published data. The past century has witnessed much progress in the materials for RVOT reconstruction. However, lack of growth potential is the dilemma for small-sized conduits. Tissue-engineering based on cell-free scaffolds is the most promising technology to obtain the ideal conduit.

**Summary:** No conduit has proved to have lifelong durability in RVOT position. We are far from the ideal, but we are not in a state of emergency. In-depth clinical research as well as innovation in material science are needed to help improve the durability of the conduits used in infants and young children.

## Introduction

Despite developments in materials science, we still do not have an ideal valved conduit for right ventricular outflow tract (RVOT) reconstruction (1). Currently, no conduit can meet the long-term functional requirements, especially the small-sized (≤16 mm) conduit used in infants and young children. In addition, the standard surgical timing of critical congenital heart disease has shifted to an earlier stage, with most patients having surgical treatment in the first year after birth (2). Furthermore, the Ross operation has been widely applied in pediatric patients (3). The demand for small-sized conduits has been ever-increasing.

Cryopreserved pulmonary homograft had been regarded as the gold standard materials for RVOT reconstruction for a long period of time. However, in addition to its inadequate durability (4–6), small-sized homograft is in a severe shortage. Besides, the second homograft usually performed much worse than that of the first one in the same patient (7). These issues have pushed us to explore alternative materials for RVOT reconstruction. One way to help deal with the shortage of small-sized conduits is the down-sized technique (8). We were pleased to find that this technique does not affect the the function and durability of the conduits (9, 10). There is no doubt that the bovine jugular vein conduit (BJVC), usually known as Contegra^®^ conduit (Medtronic Inc., Minneapolis, Minnesota, USA), has achieved great success. It showed comparable (11, 12) or even better (13–15) performance than homograft. Still, the durability of BJVC is very poor in infants (16). To make matters worse, the Contegra conduit had not been authorized for profit upon sale until 2013 and is still not available in China and other countries. Recently, the Japanese approach of hand-sewn expanded polytetrafluoroethylene (ePTFE) valved conduit has attracted much attention, with excellent long-term outcomes demonstrated in multi-center studies where more than 90% small-sized conduits were free from explantation at 5 years (17–19). However, very limited numbers of heart centers outside of Japan reported the application of this conduit, perhaps due to the inconsistent quality control of a hand-sewn valve.

An ideal conduit should be one with permanent physiological functions and with growth potential adapting to the patient's somatic growth. That is, the conduit should be “alive” in the host. The development of tissue engineering technology provides us with the opportunity to realize the ideal. In this review, we focus on the history evolution and current situation of small-sized conduits (≤16 mm) for RVOT reconstruction in infants and young children. The difficulties as well as the future direction will be discussed to provide assistance for the development of pulmonary valved conduits.

## History

**Figure 1:**
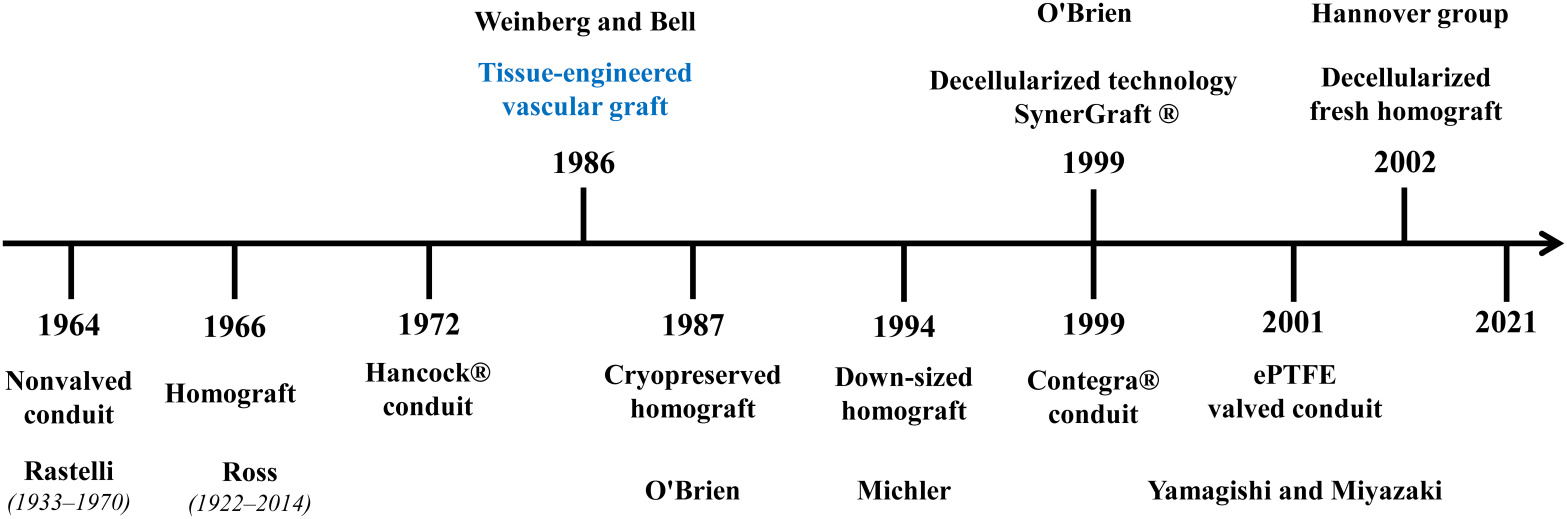
Development of small-sized conduits for RVOT reconstruction. The commonly used conduit materials for RVOT reconstruction were listed in the figure in chronological order with the founder(s) (if any). Tissue engineered vascular graft has the potential to achieve an ideal conduit, but it is still in development.

The history timeline of available small-sized conduits is shown in Figure 1. In the development of conduit materials for RVOT reconstruction, the valveless conduit was the first attempt and is worthy of being mentioned. It was reported as serving as the RVOT material in REV (Réparation à l'Etage Ventriculaire) operations and truncus arteriosus repair (20, 21). However, most centers now prefer a valved conduit due to the undoubted importance of functional pulmonary valve.

The homograft conduit constitutes a crucial part in the history of pulmonary conduits (22). It is the first and most widely used valved conduit for ROVT reconstruction. During the past half-century, the improvement of processing and preserving technology has facilitated superior durability for homograft (8, 23, 24). Down-sized technique makes up for the shortage of homograft for young children and has achieved satisfactory durability. But none of them can achieve lifelong durability and therefore serve as a palliative treatment for young children. Later on, many xenografts were introduced for RVOT reconstruction, but only a few of them are suitable for infants and young children. The Hancock^®^ conduit (Medtronic Inc., Minneapolis, Minnesota, USA) is the first commercial xenograft which consisted of porcine valve sutured into a Dacron conduit. The Contegra conduit (Medtronic Inc.) is one of the most successful xenografts which was mainly treated with glutaraldehyde. The xenografts were easier to obtain with much lower cost than homografts, but still, without much improvement in durability, particularly for young children. The Japanese approach of hand-sewn ePTFE valves is a great event in the history of conduits (25). Japanese researchers have established a mature system for the construction of hand-made ePTFE valved conduits and achieved amazing clinical outcomes.

Throughout history, we have achieved great success in conduit materials' evolution for RVOT reconstruction in young children. Currently, the available conduits (Table 1) can meet the clinical needs in most countries, and re-thoracotomy for conduit replacement is no longer a high-risk operation for cardiac surgeons. Moreover, trans-catheter pulmonary valve replacement (TPVR) has been proven to be a safe and effective therapeutic for failed pulmonary conduits (34). Therefore, our current task is to improve the durability and prolong the life of the valved conduit for RVOT reconstruction. We are far from the ideal, but we are not in a state of emergency.

**Table 1:** Clinical results of small-sized valved conduits for right ventricular outflow tract reconstruction in infants and young children.

**Authors**	**Conduit**	**No. of patients**	**Age/Weight/Conduit size** ^**a**^	**Durability**
Kaza et al. (Children's Hospital Boston, 2009). (26)	Pul. homograft (non-Ross)	38	** <30 d**./NA/NA	FF reintervention: ~25% at 5 yr., 6.7% at 14 yr.^**b**^
	Aortic homograft (non-ross)	34	** <30 d**./NA/NA	FF reintervention: ~10% at 5 yr., 0 at 10 yr.
Brown et al. (Indiana University School of Medicine, 2005). (5)	Homograft (non-Ross)	57	** <1 yr**./NA/NA	FF failure: 42% at 5 yr., 34% at 15 yr.
Mainwaring et al. (Lucile Packard Children's Hospital. 2015). (27)	Aortic homograft (non-Ross)	25	** <1 yr**./NA/ 8–11 mm	MTR: 1.3 yr.
		45	** <1 yr**./NA/ 12–14 mm	MTR: 3.3 yr.
		33	** <1 yr**./NA/ 15–17 mm	MTR: 4.2 yr.
Nelson et al. (University of Michigan Medical School. 2015). (28)	Homograft (ross)	44	** <1 yr**./NA/14.0 mm	FF reintervention: 52% at 5 yr., 19% at 15 yr.
Cleuziout et al. (German Heart Centre Munich. 2016). (9)	Homograft	70	17.7 mo./ ** <14 kg**/14.8 mm	FF reintervention: 79% at 5 yr., 61% at 10 yr.
	Down-sized homograft	82	16.2 mo./ ** <14 kg**/14.6 mm	FF reintervention: 87% at 5 yr., 69% at 10 yr.
Romeo et al. (Erasmus University Medical Center. 2018). (10)	Homograft	28	** <2 yr**./6.1 kg/ 16.1 mm	FF replacement: 94% at 5 yr., 71% at 10 yr.
	Down-sized homograft	19	** <2 yr**./6.0 kg/ 14.7 mm	FF replacement: 95% at 5 yr., 82% at 10 yr.
Francois et al. (University Hospital Ghent. 2018). (29)	Pul. homograft	40	** <3 yr**./11.9 kg/17.2 mm	FF SVD: 68% at 10 yr. MTR: 7.6 yr.
	Down-sized homograft	17	** <3 yr**./7.4 kg/ 16.3 mm	FF SVD: 42% at 10 yr. MTR: 8.3 yr.
	BJVC	24	** <3 yr**./8.0 kg/15.8 mm	FF SVD: 20% at 10 yr. MTR: 5.6 yr.
Poynter et al. (CHSS member institutions, 2013). (13)	BJVC and homograft	429	** <2 yr**./ 4.9 kg/NA	FF replacement: 53% at 8 yr.
Sandica et al. (Two centers in German. 2016). (30)	BJVC	97	** <1 yr**./NA/NA	FF replacement: ~20% at 5 yr., ~5% at 10 yr.
	Homograft	25	** <1 yr**./NA/NA	FF replacement: ~0 at 5 yr.
Patel et al. (Indiana University School of Medicine, 2018). (16)	BJVC	65	** <1 yr**./5.1 kg/13.1 mm	FF replacement: 53% at 5 yr., 15% at 10 yr.
Falchetti et al. (Queen Fabiola Children's University Hospital, 2019). (12)	BJVC	30	** <30 d./**3.3 kg/12 mm	FF reoperation: ~60% at 5 yr., ~20% at 10 yr.
	Homograft	23	** <30 d**./2.8 kg/9–14 mm	FF reoperation: ~70% at 5 yr., ~30% at 10 yr.
Buckley et al. (Multicenters in the United States, 2019). (31)	BJVC (TA)	55	NA/NA/**12 mm**	MTR: 20 mo
	Pul. homograft (TA)	83	NA/NA/**9–11 mm**	MTR: 23 mo
	Aortic homograft (TA)	53	NA/NA/**9–11 mm**	MTR: 26 mo
Herrmann et al. (Indiana University School of Medicine, 2020). (14)	BJVC (TA)	36	**33 d./3.2 kg/12 mm**	FF reoperation: ~65% at 5 yr., ~25% at 10 yr.
	Pul. homograft (TA)	41	**33 d./3.2 kg/12 mm**	FF reoperation: <50% at 5 yr., <10% at 10 yr.
	Aortic homograft (TA)	14	**33 d./3.2 kg/12 mm**	FF reoperation: ~35% at 3 yr., ~0 at 7 yr.
Yamashita et al. (Multicenters in Japan, 2016). (18)	ePTFE valved conduit	303	18 mo./8.4 kg/**≤16 mm**	FF replacement: 90% at 5 yr.
Miyazaki et al. (Multicenters in Japan, 2018). (19)	ePTFE valved conduit	292	** <2 yr**./12.6kg/NA	FF replacement: 90% at 5 yr., 74% at 10 yr.
	ePTFE valved conduit	400	NA/NA/**≤16 mm**	FF replacement: 92% at 5 yr., 76% at 10 yr.
Mercer et al. (Children's Hospital of Pittsburgh, 2018). (32)	Bicuspid ePTFE valved conduit	39	** <2 yr**./5.7 kg/11.8 mm	Incidence of replacement: 17% at 1 yr., 55% at 5 yr.
	Homograft	26	** <2 yr**./5.6 kg/11.2 mm	Incidence of replacement: 23% at 1 yr., 56% at 5 yr.
Seese et al. (Children's Hospital of Pittsburgh, 2020). (1)	Bicuspid ePTFE valved conduit (TA)	18	** <30 d**./NA/11 mm	FF replacement: 82% at 5 yr., 27% at 10 yr.
	Homograft (TA)	7	** <30 d**./NA/10 mm	FF replacement: 71% at 5 yr., 29% at 10 yr.
Vitanova et al. (German Heart Centre Munich, 2014). (11)	Hancock conduit	48	** <1 yr**./4.3 kg/ ≤ 14 mm	FF replacement: 54% at 5 yr., 20% at 10 yr.
	Homograft	62	** <1 yr**./4.6 kg/ ≤ 16 mm	FF replacement: 69% at 5 yr., 38% at 10 yr.
	BJVC	35	** <1 yr**./4.2 kg/ ≤ 16 mm	FF replacement: 59% at 5 yr., 38% at 10 yr.
Ruzmetov et al. (Children's Hospital of Illinois, 2012). (33)	Decellularized homograft	6	** <1 yr**./NA/NA	FF replacement: 67% at 10 yr.
	Homograft	17	** <1 yr**./NA/NA	FF replacement: 25% at 10 yr.

a *Values of age/weight/conduit size are mean or median or range. The bold words indicate the main characteristics of the study cohort*.

b *“~” indicates the value was obtained according to the survival curve in the literature*.

*BJVC, bovine jugular vein conduit; CHSS, the Congenital Heart Surgeons Society; ePTFE, expanded polytetrafluoroethylene; FF, freedom from; MTR, mean/median time to replace; NA, not available; Pul., pulmonary; SVD, structural valve degeneration; TA, truncus arteriosus*.

## Clinical Outcomes of Available Conduits

### Homograft

Generally, cryopreserved homograft conduits perform much better in adults than in childrens, as well as in Ross operations than in non-Ross operations (35, 36). As detailed in Table 1, only a few studies analyzed the durability in infant or young children subgroups.

Kaza et al. found only 6.7% of pulmonary homograft conduits were free from reintervention at 14 years in newborns who underwent surgical repair of tetralogy of Fallot and truncus arteriosus. On the other hand, all aortic homograft conduits were replaced within 10 years (37). Brown et al. reported their experience of homograft in 117 non-Ross operations, in which nearly half of the patients were infants (*n* = 57, 49%) (5). The freedom from homograft failure (defined as reintervention or patient death) was 42% at 5 years and 34% at 15 years. Another study focused on the fate of small-sized homograft in non-Ross operations revealed that almost all homograft conduits smaller than 12 mm had to be replaced within 3 years (26). The 5-year freedom from replacement was approximately 10% and 30% respectively for conduits with diameters of 12–14 and 15–17 mm (26). The long-term results of cryopreserved homograft implanted in Ross operations in infants were reported by Nelson et al. (27). The freedom from homograft reintervention for 44 infants in this cohort was 52% at 5 years and 19% at 15 years.

The down-sized technique was first described by Michler et al. in 1994, obtaining a bicuspid valved conduit by removing one of the three valve leaflets and the attached wall (8). The largest study cohort of down-sized homograft conduits came from the German Heart Centre, Munich (9). Eighty-two patients receiving a down-sized homograft were compared to 70 patients receiving normal small-sized homograft. Both the mean conduit diameter and the freedom from replacement were comparable in the two groups (14.8 vs. 14.6 mm; 69 vs. 61% at 10 years). Romeo et al. reported similar results in patients under 2-years-old (10). More recently, Francois et al. finished another study focused on the comparison between down-sized homograft and other conduits in patients under 3-years-old (28). The freedom from structural valve degeneration (SVD, defined as conduit dysfunction or replacement) at 10 years was 68, 42, and 31% for pulmonary homograft, down-sized homograft, and aortic homograft, respectively. Multivariable analysis confirmed the down-sized technique did not increase the risk of SVD.

There is no doubt that the cryopreserved homograft is immunogenic as the cryopreserved technology retained lots of allogeneic cells (29). The graft-related immune response plays an important role in the failure of cryopreserved homograft in young children (38). A response to the immunogenicity was decellularized technology (SynerGraft, CryoLife, Kennesaw, GA) proposed by O'Brien in 1999 (24). Decellularization preserves the extracellular matrix scaffold while greatly eliminating the immunogenicity from allogeneic cells, resulting in a milder immune reaction in the host. Ruzmetov *et al*. compared decellularized and standard cryopreserved homograft inserted primarily in patients <1 year old, as part of their single-institution study (39). Both the 10-year freedom from replacement and reintervention of the decellularized homograft trended better. A multicenter study demonstrated significantly higher durability of the SynerGraft homograft conduit compared with that of standard cryopreserved homograft (40). The decellularized fresh homografts developed by the Hannover group were first applied after pre-seeding of endothelial progenitor cells for two pediatric patients in 2002 (41). The conduits have been widely used in Europe (the ESPOIR Trial) without pre-seeding of stem cells (30, 33, 42). The latest long-term follow-up results showed significantly better freedom from explantation and less structural valve degeneration when matched to cryopreserved homograft and BJVC (42). The decellularized homograft is expected to be a better choice for infants and young children, but needs more evidence from large samples with longer follow-ups.

### Bovine Jugular Vein Conduit

The BJVC, usually known as Contegra^®^ conduit, was introduced as a substitute to homograft. Patel and Brown et al. reported the freedom from BJVC explantation was 53% at 5 years and 15% at 10 years for infants (16). The mean time to conduit failure was 5.2 years in the infants subgroup and 5.8 years in the small-sized conduits (12–14 mm) subgroup (16). Germany took the lead in clinical use of BJVC. Later on, a series of multicenter studies were carried out. The latest multicenter study revealed that the BJVC performed much better than homograft in infants. In fact, about 90% of the BJVC became dysfunctional 10 years after implantation, while a similar outcome only took 4 years in homograft recipients (43). The Congenital Heart Surgeons Society reported a series of high-impact multicenter studies on the conduits for RVOT reconstruction in young children (13, 31, 44). Their recommendation of oversizing conduits with z-scores between +1 and +3 is still widely acknowledged today (31). Two other studies also demonstrated that BJVC has better performance than homograft in infants and young children (13, 44).

Recently, the durability of BJVC and homograft have been further compared in strictly matched newborns (12, 14, 44, 45). One multicenter study included three groups using different conduits (83 pulmonary homograft conduits, 53 aortic homograft conduits, and 55 BJVCs) in truncus arteriosus repair (45). The study found that, although the three groups showed similar portions of replacement, the reintervention of BJVC was significantly less than homograft conduit. A similar conclusion was reported in another long-term longitudinal study of patients with truncus arteriosus (14). More than 20% of BJVC was free from reoperation at 10 years, while less than 10% of pulmonary homograft conduits and no aortic homograft conduit were free from reintervention (14).

As detailed in Table 1, the highest rate of 10-year freedom from BJVC replacement was approximately 25% in young children (12, 14). Although the results from different studies were not consistent, the BJVC showed similar or even better durability than that of cryopreserved homograft for RVOT reconstruction. However, the BJVC has greater incidence of late endocarditis after being implanted, with a rate of 4.7% to 9.9% reported in previous studies (16, 46–48). The endocarditis seems to be a unique situation that occurs in glutaraldehyde-treated bovine jugular vein products (49). The reasons are still unclear, but the infection will definitely exacerbate the failure of the conduit.

### The Hand-Made ePTFE Valved Conduit

The 0.1 mm thick ePTFE has been employed as the pulmonary valve material since the 1990s (50, 51). Yamagishi, Miyazaki, and colleagues carried out a series of impressive technological innovations in Japan, contributing to the hand-made ePTFE valved conduit which consisted of three bulging sinuses and tricuspid fan-shaped valve (17, 52).

In Japan, the ePTFE valve is preferred in the form of a conduit instead of the originally designed valved patch (53), as they found that even the small-sized (≤ 16 mm) conduits had satisfactory durability (18). A nationwide multicenter study revealed that 90.1% small-sized ePTFE valved conduits were free from replacement at 5 years (18). More recently, another nationwide multicenter study in Japan incorporated 292 patients of <2 years of age and found that the freedom from replacement was 90% at 5 years, 74% at 10 years, and 67 % at 12 years (19). When examined by conduit diameter, the freedom from small-sized conduit replacement was 76% at 10 years (19).

The clinical results from Japan were excellent. However, this artificial conduit was only used in a handful of heart centers outside of Japan (54, 55). In the last decade, the application of 0.1 mm thick ePTFE for RVOT reconstruction has gradually increased in the form of bicuspid valves directly sutured into RVOT (56, 57), or bicuspid/tricuspid valved conduits without bulging sinuses (32, 58). Mercer et al. reported a similar performance for bicuspid ePTFE valved conduits with homograft conduits in patients <2 years of age (59). Another study from this team focusing on neonatal truncus arteriosus repair also showed similar durability, with 10-year freedom from replacement of 27.3 and 28.5% respectively for ePTFE valved conduit and homograft conduit (60).

Despite remaining controversial, the introduction of hand-sewn ePTFE valved conduits for RVOT reconstruction is a great innovation. Current data show that it has a high tendency to become another successful substitute for pulmonary conduit.

### Other Conduits

The Hancock^®^ conduit (Medtronic Inc.) is the oldest xenogeneic valved conduit which is still in use. Vitanova et al. reported the freedom from Hancock conduit replacement of 53.8% at 5 years and 20.3% at 10 years, which was similar to that of homograft and BJVC in young children (11). To the best of our knowledge, other xenografts, including the Carpentier-Edwards conduit (Edwards Lifesciences, Irvine, California, USA), Freestyle porcine aortic root (Medtronic Inc.), and stented bioprosthetic valves, were rarely reported as being used in infants and young children. In the past 10 years, TPVR has emerged as an alternative to open-heart surgery in selected patients (61). This technology has not been applied in infants or young children. However, conduits like Hancock have been identified to be more favorable for future TPVR and thus have been more used.

Another alternative allograft for RVOT reconstruction in infants is valved femoral vein homograft (FVH). Sinha et al. reported the first case of a 2-month-old girl diagnosed with Fallot with pulmonary atresia in 2008 (62). By the end of 2012, they had implanted a total of 20 FVHs in neonates and infants and found a lower rate of both reintervention and reoperation compared with homograft in the short- to mid-term (63). Theoretically, FVH has thinner walls and fewer donor cell components, which is beneficial to the infiltration and growth of host cells after implantation. Carreon et al. found that 1 year after being implanted as a pulmonary conduit, the venous walls underwent remodeling with only minimal inflammation and calcification, and the venous valve leaflets were relatively spared from hyperplasia with preserved function (64). However, more studies are required to explore the fate of FVH in RVOT position.

Tissue engineering valved conduits is a perfect technology assumption, which is supposed to achieve lifelong functional requirements through *in-vitro* cell seeding and culturing, bio-printing, or *in-vivo* regeneration approaches (65). It seems to be more feasible to induce remodeling *in vivo* based on the cell-free natural scaffolds or synthetic biodegradable materials, other than *in-vitro* cell culturing (66). Significant efforts have been put into the synthetic biodegradable valved conduit scaffolds (67, 68). Bennink et al. recently reported the outcomes of an animal experiment of a synthetic biodegradable conduit processed by electrospinning (69). Over a 1 year follow-up period, the pulmonary conduits were progressively remolded and degraded with stable functionality in the sheep model. The CorMatrix^®^ conduit (CorMatrix Cardiovascular, Inc., Roswell, USA) is a biodegradable natural scaffold composed of porcine small intestinal submucosa extracellular matrix (70). Unfortunately, it failed to remodel as a pulmonary conduit before the valve degeneration in both porcine model and xenogeneic animal model (71, 72). Ghorbel et al. seeded the mesenchymal stem cell-derived vascular smooth muscle cells *in vitro* into the CorMatrix conduit (73). Six months after being implanted to reconstruct the left pulmonary artery in piglets, the cellularized-CorMatrix grafts were remolded with homogeneous endothelium covered by multi-layered muscular media (73).

In our institution, we have been devoted to research on BJVC since 2002. During this time, we successfully developed a novel decellularized and photo-oxidatively crosslinked BJVC (74–76). This conduit is a cell-free xenogeneic scaffold conducive to host cells' attachment and growth. After a series of strict large animal experiments, it has been put into clinical application in several centers in China. The 10-year freedom from conduit replacement and catheter-based reintervention were 94.7% and 52.9%, respectively, for children ≤3 years old (77).

## Future Direction

An ideal pulmonary conduit for reconstruction of RVOT should be “alive”: composed of viable host cells, equipped with stable functionality, and able to grow with the patient's somatic growth synchronously. Tissue-engineered conduits are expected to meet all of the requirements to make it an ideal conduit. After decades of exploration, however, this technology is still in its infancy.

It seems that we have not made a substantial breakthrough in conduit materials for RVOT reconstruction, especially in conduits for infants and young children. Lacking in growth potential is an issue that seems insurmountable at present. For adults, we actually do not need an “alive” conduit due to the non-obvious somatic alterations, as long as one conduit can maintain long-term structure stability and functionality. The ePTFE valved conduit therefore may provide greater durability than biological grafts in adults, as it is biologically inert and resistant to immunological degeneration. For children, several studies concluded that somatic outgrowth is not the core reason for the failure of pulmonary conduit (6, 7), that is, the conduit has failed even before its mismatch. Children may also benefit from biologically inert conduits.

The development of novel pulmonary valved conduits based on tissue engineering technology is the most promising way to tackle the dilemma. In addition, modification based on the currently available conduits, such as the preservation technique for homograft, anti-calcification, and anti-infection modification of glutaraldehyde-treated BJVC, may help to achieve better durability. In addition, large multicenter studies, rigorous meta-analysis focused on specific concerns, and comparisons between different conduits in certain conditions might be helpful to establish unified standards for pulmonary valved conduit implantation.

## Conclusion

We still do not have an ideal valved conduit for RVOT reconstruction in infants and young children. But fortunately, the available small-sized conduits can meet most of the clinical demand. Homograft and BJVC are still reliable choices for young children. Hand-made ePTFE valved conduits have very satisfactory performance based on currently limited reported data. Innovation in material science and more in-depth clinical studies in this special field are required to get better durability. Tissue engineering technology based on cell-free scaffolds is still the most promising way to obtain an ideal “alive” valved conduit, but there is still a long way to go.

## Author Contributions

ZW make substantial contributions to conception and design, and give final approval of the version to be submitted. TQ drafted and revised the manuscript. HY, CC, YL, TL, and CH participate in revising the manuscript critically for important intellectual content. All the authors have contributed significantly to the review.

## Conflict of Interest

The authors declare that the research was conducted in the absence of any commercial or financial relationships that could be construed as a potential conflict of interest.

## Publisher's Note

All claims expressed in this article are solely those of the authors and do not necessarily represent those of their affiliated organizations, or those of the publisher, the editors and the reviewers. Any product that may be evaluated in this article, or claim that may be made by its manufacturer, is not guaranteed or endorsed by the publisher.
